# Codon-triplet context unveils unique features of the *Candida albicans *protein coding genome

**DOI:** 10.1186/1471-2164-8-444

**Published:** 2007-11-29

**Authors:** Gabriela R Moura, José P Lousado, Miguel Pinheiro, Laura Carreto, Raquel M Silva, José L Oliveira, Manuel AS Santos

**Affiliations:** 1 Department of Biology and CESAM. University of Aveiro, 3810-193 Aveiro, Portugal; 2 ESTGL, Polytechnic Institute of Viseu, 5100-074 Lamego, Portugal; 3 Institute of Electronics and Telematics Engineering (IEETA). University of Aveiro, 3810-193 Aveiro, Portugal

## Abstract

**Background:**

The evolutionary forces that determine the arrangement of synonymous codons within open reading frames and fine tune mRNA translation efficiency are not yet understood. In order to tackle this question we have carried out a large scale study of codon-triplet contexts in 11 fungal species to unravel associations or relationships between codons present at the ribosome A-, P- and E-sites during each decoding cycle.

**Results:**

Our analysis unveiled high bias within the context of codon-triplets, in particular strong preference for triplets of identical codons. We have also identified a surprisingly large number of codon-triplet combinations that vanished from fungal ORFeomes. *Candida albicans *exacerbated these features, showed an unbalanced tRNA population for decoding its pool of codons and used near-cognate decoding for a large set of codons, suggesting that unique evolutionary forces shaped the evolution of its ORFeome.

**Conclusion:**

We have developed bioinformatics tools for large-scale analysis of codon-triplet contexts. These algorithms identified codon-triplets context biases, allowed for large scale comparative codon-triplet analysis, and identified rules governing codon-triplet context. They could also detect alterations to the standard genetic code.

## Background

The degeneracy of the genetic code allows synthesis of identical proteins from mRNAs with rather different primary structures. This bias in synonymous codon usage is linked to tRNA abundance, codon-pair context effects, genome G + C pressure, the strength of codon-anticodon interactions, and to other DNA replication, transcription and mRNA translation biases [1-5]. Interestingly, codon-pair context fine tunes mRNA decoding efficiency [6-9]. For example, in *E. coli*, 3'context alteration from G to U in the insertion sequence IS911 (A-AAA-AAG) increases frameshifting from 10% to 60% [10], while, intriguingly, the over-represented ACG-CUG codon-pair is translated slower than the under-represented synonymous codon-pair ACC-CUG [9].

Those context effects suggest that codon-pairs are important modulators of mRNA translation accuracy and speed. However, codon-pairs cannot reflect the full bias imposed by the translational machinery on mRNA primary structure since the ribosome has 3 rather than 2 decoding sites, namely A-, P- and E-sites [11]. The A- and P-sites are directly involved in aminoacyl-tRNA (aa-tRNA) selection and translocation and, for these reasons, it is not surprising that codon-pair context influences protein synthesis fidelity. From a structural perspective, the role of the E-site, which is occupied by deacylated tRNA during exit from the ribosome [12], on mRNA decoding speed and accuracy is not so clear. However, E-site occupation does influence decoding fidelity by changing allosterically the affinity of the A-site during selection of in-coming aa-tRNAs [13-15]. This allosteric interaction between the E- and A- sites, plus ribosome crystallography and cryo-EM studies [16], provide strong functional and structural evidence for a critical role of the 3 tRNAs accommodated in the ribosome in decoding efficiency. In other words, the E-site is more than just an exit site for deacylated tRNAs from the ribosome. Hence, codon-triplets present in the ribosome A-, P- and E-sites are expected to play an important role in the accuracy and efficiency of mRNA translation. If so, like codon-pair context, codon-triplet context should be biased. This hypothesis is supported by the observation that non-programmed translational frameshifting and programmed translational events involve more than two consecutive codons (e.g. [8,10]).

In a previous study, we have developed software and statistical methodologies for analysis of codon-pair contexts and have identified general rules that govern such context [4]. In here, we have extended those studies to the analyses of codon-triplets context, using several fungal ORFeomes as model systems. This study produced very large data sets and posed significant computational challenges, which prompted the development of a dedicated database for data storage and tools for data mining. We show for the first time that context of codon-triplets is highly biased and species specific and we discuss the implications of trinucleotide repeats for codon-triplets context. We also explain how our approach can be used to identify non-standard mRNA decoding events and alterations to the genetic code.

## Results

### Tools to study codon-triplets

**Figure 1:**
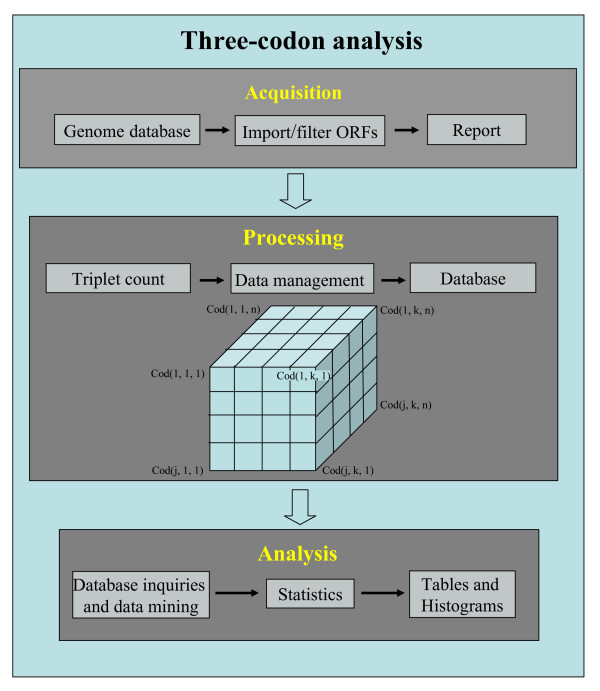
**Schematic representation of the bioinformatics system**. Gene sequences were downloaded from genome databases (Table 1) and filtered into a local database to eliminate false Open Reading Frames. Sequences were then processed by counting all codon-triplets, excluding the first and the last ones of each ORF, which have specific translation initiation and termination contexts. These data were transferred to a 3-dimensional 61 × 61 × 61 matrix and were saved as a Microsoft Access Database file. The processed data were then analyzed using Weka-3 data mining tools [19] and direct database queries. This methodology allowed us to handle very large data sets and identify differences in codon-triplet context between fungal species. These differences were finally subjected to statistical analyses.

We have implemented computational algorithms and data storage facilities for comparative genomics of codon-triplet analysis in fungal genomes (Figure 1). The algorithm developed simulates the ribosome during decoding by reading Open Reading Frames (ORFs), from the ATG initiation codon, and moving the reading window three nucleotides at a time until a stop codon is encountered. While doing this, it memorizes all codon-triplets, which represent A-, P- and E-site codons during mRNA decoding. In this study, triplet counting was performed on complete sets of ORFs (ORFeomes), which were initially filtered to eliminate aberrant ORFs lacking ATG initiation or TAG/TGA/TAA stop codons, or containing premature stop codons or ambiguous bases (N). The first and last triplets of each ORF were not considered to avoid translation initiation and termination context effects [17,18].

Since analysis of codon triplets generates a 3-dimensional 61 × 61 × 61 matrix for each ORFeome, we have used a relational database to store the processed data (Figure 1). These large data sets were then analyzed using data mining tools [19] and direct database queries. These studies aimed at identifying major differences in triplet-codon context between the fungal ORFeomes stored in the main database (Table 1). A similar methodology was used to count amino acid triplets generated from the same ORFeome sequences. For this, codons were translated to the respective amino acids using standard genetic code rules or using non-standard decoding of the leucine-CTG codons as serine in *Candida albicans *and *Debaryomyces hansenii *[20-22]. Finally, new algorithms were implemented to count codon and amino acid repetitions on an ORFeome wide scale. All results obtained were compared with values expected for a random distribution of codons, which were calculated considering the frequencies of random distribution of individual codons or amino acids in the genomes.

**Table 1:** Data source

Species	Site/link
*A. fumigatus*	[42]
*C. albicans*	[43]
*C. glabrata*	[44]
*D. hansenii*	[45]
*E. gossypii*	[46]
*K. lactis*	[47]
*S. bayanus*	[48]
*S. cerevisiae*	[49]
*S. mikatae*	[48]
*S. paradoxus*	[48]
*S. pombe*	[50]

11 ORFeome sequences were downloaded from the web sites indicated bellow between December 2005 and February 2006. Each ORFeome was scanned for detection of invalid ORFs (see Methods), lacking ATG-start and TAA, TAG and TGA stop-codons. ORFs containing permature stop codons or undefined nucleotides (N) were also discarded from the analysis.

### Codon-triplets in fungal genomes

**Figure 2:**
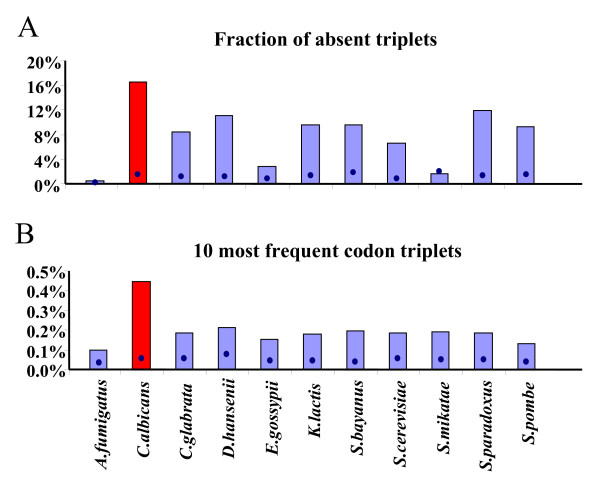
**Major differences in codon-triplet contexts in fungal genomes**. In order to characterize codon-triplet distributions in the 11 fungal species studied, we have calculated the percentage of codon-triplets that did not appear in the fungal ORFeomes (panel A). Additionally, the fraction corresponding to the 10 most frequent codon-triplets were also quantified (panel B). In both cases, *C. albicans *showed stronger bias in codon-triplet distribution than the other species. Bars represent observed percentages while blue dots indicate values expected from random codon-triplet distribution.

The tools described above permitted carrying out a comparative analysis of codon-triplets in 11 fungal ORFeomes (Table 1). Clear patterns of codon-triplets preferences and rejections were identified for each ORFeome and, as for codon-pair contexts [4], such patterns were specific of each ORFeome (Tables 2 and 3 and Additional file 1, Figure S1). This first analysis also showed that the percentage of codon-triplets that vanished from the ORFeomes was much higher than expected from random distribution of the triplets in these ORFeomes (Figure 2A). The percentage of vanished codon-triplets varied between 8 and 11% in most fungal ORFeomes, but was significantly lower in *Aspergillus fumigatus *(0.5%), *Eremothecium gossypii *(2.9%) and *Saccharomyces mikatae *(1.6%). The human pathogen *Candida albicans *had higher percentage of such triplets (16.5%) and those vanished codon-triplets were reflected also at the amino acid level, since *C. albicans *was the only species where an amino acid triplet, namely Trp-Met-Trp, was absent. Conversely, analysis of the 10 most frequent codon-triplets (Figure 2B) showed an even distribution in these fungal ORFeomes with exception of *C. albicans*, where the percentage of these abundant triplets increased more than 2-fold (0.45%). Overall, in *C. albicans *there was a clear over-representation of a subset of codon-triplets, namely (CAA-CAA-CAA), (GAA-GAA-GAA), (AAT-AAT-AAT) or (GAT-GAT-GAT) (Table 2) and strong repression of another subset, namely (GAA-AAA-AAA), (AAA-AAA-AAT), (AAA-AAA-AAA) or (TTA-AAA-AAA) (Table 3), indicating higher bias of codon-triplet usage in this human pathogen (Additional file 1, Figure S1).

**Table 2:** Ranking of the 10 most preferred codon-triplets in fungal ORFeomes

	*A.fum*	*C.alb*	*C.gla*	*D.han*	*E.gos*	*K.lac*	*S.bay*	*S.cer*	*S.mik*	*S.par*	*S.pom*
1	**AAG**	**CAA**	**GAA**	**GAA**	**CAG**	**GAA**	**GAA**	**GAA**	**GAA**	**CAA**	**GAA**
	**AAG**	**CAA**	**GAA**	**GAA**	**CAG**	**GAA**	**GAA**	**GAA**	**GAA**	**CAA**	**GAA**
	**AAG**	**CAA**	**GAA**	**GAA**	**CAG**	**GAA**	**GAA**	**GAA**	**GAA**	**CAA**	**GAA**
2	**GAG**	**GAA**	**AAG**	**GAT**	**GAG**	**CAA**	**CAA**	**GAT**	**GAT**	**GAA**	ATT
	**GAG**	**GAA**	**AAG**	**GAT**	**GAG**	**CAA**	**CAA**	**GAT**	**GAT**	**GAA**	ACT
	**GAG**	**GAA**	**AAG**	**GAT**	**GAG**	**CAA**	**CAA**	**GAT**	**GAT**	**GAA**	AGT
3	GAG	**AAT**	**GAT**	**CAA**	**AAG**	**GAT**	**AAC**	**CAA**	**CAA**	**GAT**	ACA
	AAG	**AAT**	**GAT**	**CAA**	**AAG**	**GAT**	**AAC**	**CAA**	**CAA**	**GAT**	CCA
	AAG	**AAT**	**GAT**	**CAA**	**AAG**	**GAT**	**AAC**	**CAA**	**CAA**	**GAT**	ATT
4	**CAG**	**GAT**	GAT	GAT	**GAC**	**AAG**	**CAG**	GAT	**CAG**	**AAT**	CCA
	**CAG**	**GAT**	GAA	GAA	**GAC**	**AAG**	**CAG**	GAT	**CAG**	**AAT**	ATT
	**CAG**	**GAT**	GAA	GAA	**GAC**	**AAG**	**CAG**	GAA	**CAG**	**AAT**	ACT
5	**GAA**	**AAC**	GAT	GAA	**GCG**	CAA	CAA	**AAT**	CAA	CAA	GAT
	**GAA**	**AAC**	GAT	GAT	**GCG**	CAG	CAG	**AAT**	CAG	CAG	GAA
	**GAA**	**AAC**	GAA	GAA	**GCG**	CAA	CAA	**AAT**	CAA	CAA	GAA
6	GAG	CAA	GAT	GAT	GAG	GAT	**GAT**	CAA	**AAT**	GAT	TCC
	GAG	CAG	GAA	GAT	GAC	GAA	**GAT**	CAG	**AAT**	GAA	ACA
	GAA	CAA	GAT	GAA	GAG	GAT	**GAT**	CAA	**AAT**	GAA	CCA
7	GAA	**GGT**	AAG	**AAG**	GAG	**CAG**	**AAG**	GAT	GAT	**CAG**	GAA
	GAG	**GGT**	AAG	**AAG**	CTG	**CAG**	**AAG**	GAA	GAT	**CAG**	GAT
	GAG	**GGT**	AAA	**AAG**	CTG	**CAG**	**AAG**	GAA	GAA	**CAG**	GAA
8	AAG	CAA	**CAA**	GAT	GAC	CAG	**AAT**	GAA	GAT	GAT	**GAT**
	GAG	CAA	**CAA**	GAA	GAG	CAA	**AAT**	GAT	GAA	GAT	**GAT**
	AAG	CAG	**CAA**	GAT	GAC	CAA	**AAT**	GAA	GAA	GAA	**GAT**
9	GAA	GAT	GAA	GAC	GAC	CAA	GAA	GAT	GAA	GAA	**TCT**
	GAG	GAA	GAT	GAA	GAG	CAA	GAT	GAA	GAT	GAT	**TCT**
	GAA	GAA	GAA	GAA	GAG	CAG	GAA	GAT	GAA	GAA	**TCT**
10	GAG	**CCA**	GAA	**AAT**	CTG	GAT	GAA	**CAG**	CAG	**AAG**	AGT
	GAG	**CCA**	GAA	**AAT**	CTG	GAA	GAG	**CAG**	CAA	**AAG**	TCA
	AAG	**CCA**	GAG	**AAT**	CAG	GAA	GAA	**CAG**	CAG	**AAG**	ACT

The complete set of codon-triplets present in the 11 fungal ORFeomes studied were identified and the codon-triplets were ranked according to the difference between observed and expected values (bias). The 10 most preferred codon-triplets indicate that the codon-triplets with highest positive bias are common in all fungal ORFeomes. For example, the strongly biased GAA-GAA-GAA triplet was preferred in 10 out of 11 fungal ORFeomes (underscored). Also, codon-triplets containing identical codons were frequent (bold). Interestingly, the most common feature of these preferred codon-triplets was the presence of a guanosine (G) at position 4 and 7 of the triplet (X_1_X_2_X_3_-**Y**_4_Y_5_Y_6_-**Z**_7_Z_8_Z_9_), a feature which was common to half of the codon-triplets indicated below.

**Table 3:** Ranking of the 10 most rejected codon-triplets in fungal ORfeomes

	*A.fum*	*C.alb*	*C.gla*	*D.han*	*E.gos*	*K.lac*	*S.bay*	*S.cer*	*S.mik*	*S.par*	*S.pom*
1	GAC	GAA	GAA	GAA	GAT	GAA	AAA	GAA	GAA	AAA	GAA
	GCT	AAA	AAA	AAA	AAG	AAA	GAT	GAT	GAT	GAT	CCT
	AAG	AAA	AAG	AAA	GAG	AAA	AAG	AAG	AAG	AAG	AAA
2	GAC	AAA	AAA	AAA	GAC	GAA	GAT	AAA	AAT	ATT	GAA
	GAT	AAA	AAA	AAA	GAT	AAA	AAG	AAA	ATT	AAA	GAT
	AAG	AAT	AAG	AAA	AAG	AAG	GAA	GAA	AAA	AAA	AAA
3	GCT	AAA	GAA	GAA	GAG	AAA	GAA	GAT	ATT	GAA	AAA
	AAG	AAA	AAA	AAA	GAT	AAA	AAA	CAA	AAA	GAT	TTT
	GAC	AAA	AAA	AAT	AAG	GAA	GGT	AAA	AAA	AAG	AAA
4	GAG	TTA	GAA	AAA	GAG	AAA	GAT	AAA	AAA	TTG	ATT
	GCT	AAA	GAT	AAA	GAT	AAA	CAA	AAA	ATT	TTT	AAA
	AAG	AAA	AAG	AAT	CAG	AAG	AAA	GAT	AAA	AAA	ATT
5	GAT	CAA	GAT	AAA	AAG	AAA	AAA	GAA	GAT	AAG	GAA
	AAG	AAA	AAG	AAA	GAT	AAA	AAG	TTT	CAA	TCT	CTT
	GCC	AAA	GGT	AAG	CAG	AAA	TTG	AAA	AAA	GAA	AAA
6	GCT	GAA	AAT	GAA	GAT	GAA	GAA	AAA	ATT	AAA	ATT
	AAG	AAA	AAG	AAA	AAG	GAT	AAA	TCT	ATT	ATT	TTT
	TTC	AAT	GGT	AAG	CAG	AAA	AAA	GAA	AAA	AAA	TTT
7	GAT	AAA	AAG	GAA	AAG	GAA	GAT	GAT	AAA	GAT	GAA
	AAG	AAA	GGT	AAA	GAT	GAT	AAG	AAA	GAT	AAG	GAT
	TTC	GAA	GAT	AAC	CTG	AAG	GAT	GGT	AAG	TCT	AAG
8	ATT	TCA	AAC	CAA	TTC	GAA	GAA	AAA	GAA	GAA	GAA
	AAG	AAA	GAA	AAA	GCG	AAA	TTT	AAA	TTT	GAT	ATT
	GAG	AAA	AAA	AAA	GAG	AAT	AAG	CAA	AAG	AAA	AAA
9	GAG	TTA	AAC	AAT	GAG	GAA	AAA	AAA	AAT	ATT	ATT
	CCT	AAA	GAT	TTT	TCT	AAA	AAA	GAT	TTT	AAA	AAA
	AAG	AAT	AAG	TTA	AAG	GAT	GAT	AAT	AAA	ATT	GAT
10	GAT	CCA	GAT	GAA	TTT	GAT	AAA	AAT	AAA	AAA	TTT
	AAG	AAA	AAG	CCA	AAG	AAA	AAA	CAA	ATT	GAT	AAA
	GAG	AAA	GAA	GAT	GAG	AAA	GAA	AAA	AAT	CAA	GTT

The complete set of codon-triplets present in the 11 fungal ORFeomes studied were identified and the codon-triplet contexts were ranked according to the difference between observed and expected values (bias). This group of codon-triplets can be divided according to the presence or absence of runs of more than 5 consecutive adenosines (underlined). Interestingly, the most common feature of these strongly repressed codon triplets was the presence of an adenosine (A) at position 4 and 7 of the triplet (X_1_X_2_X_3_-**Y**_4_Y_5_Y_6_-**Z**_7_Z_8_Z_9_), a feature that appears in one third of the codon-triplets shown below.

**Figure 3:**
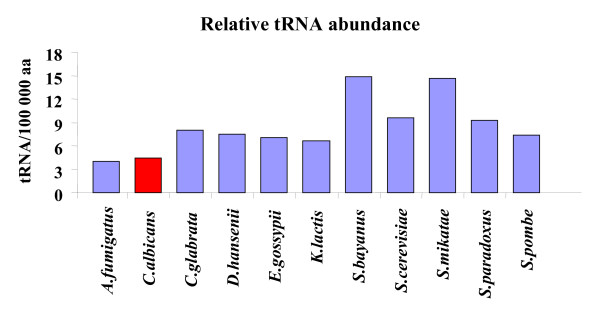
**Relative tRNA gene copy number is lower in *C. albicans *than in other fungi**. The tRNA gene copy number was determined for each tRNA isoacceptor using tRNAscan-SE [40], and the gene copy number of each group of isoacceptors was summed and divided by the number of times the respective amino acid was present in each ORFeome. In order to carry out comparisons between ORFeomes, data obtained for individual amino acids was averaged into a single column for each organism. Values are presented as tRNA gene copy number per 100 000 cognate amino acids. *A. fumigatus *and *C. albicans *have the lowest tRNA gene copy number (tRNA abundance) per aa, while *C. bayanus *and *S. mikatae *have the highest.

Since codon-triplet choice was an ORFeome specific feature that could influence mRNA decoding efficiency (see above), the stronger bias found in *C. albicans *prompted the question of whether it could be linked to mRNA translation. In order to shed new light into this question, codon usage bias and tRNA gene copy number, which provides direct indication of tRNA expression level, were determined at an ORFeome scale for all ORFeomes analyzed (Table 4). In *C. albicans*, there were fewer tRNA genes (131), but the total number of codons was the second largest (2 939 109) of the ORFeomes set. Consequently, the relative tRNA abundance (given by gene copy number) per codon (or per amino acid) was lower in *C. albicans *than in the other fungi (Figure 3). For example, the tRNA^Asn ^gene had 4 copies in *C. albicans *and between 4 and 10 copies in the other species, but the total number of Asn codons was highest in *C. albicans *(Total Asn codons = 201 917) (data not shown). In order to determine whether this relative decrease in tRNA gene copy number unbalanced tRNA abundance and codon usage, we have calculated the relative synonymous codon usage (RSCU) values for the entire set of codons (Additional file 2, Table S1), and the relative tRNA isoacceptor usage (RIU) values. The later is equivalent to RSCU values [23] but, since it was calculated using tRNA gene copy number, it informed about tRNA abundance bias. That is, different RIU values in a group of isoacceptors indicated differences in cellular levels of these tRNAs. By restricting the quotient RSCU/RIU to pairs of cognate-codons and cognate-tRNAs, we were able to calculate a Decoding Adaptation Quotient (DAQ = RSCU/RIU) by averaging the quotients obtained for each codon/tRNA pair (see Methods). DAQ values close to 1 indicated that codon usage and tRNA gene copy number (tRNA abundance) were well matched (high correlation), as was the case for *A. fumigatus*, *C. glabrata*, *E. gossypii*, *K. lactis*, *S. paradoxus *or *S. pombe *(Additional file 1, Figure S2). Organisms with DAQ>>1 often used rare tRNAs to decode frequently used codons as was the case for *D. hansenii*, *S. bayanus*, *S. cerevisiae *or *S. mikatae*. Interestingly, *C. albicans *originated the lowest DAQ value (0.841) of all fungi studied (Additional file 1, Figure S2), indicating that this fungal pathogen prefers codons that are decoded by near-cognate rather than cognate tRNAs. The divergence of decoding preferences between *C. albicans *and the other fungi can be clearly exemplified for Asn codons (AAC and AAU). All fungi analyzed decode both codons using a single tRNA (tRNA_GUU_^Asn^) and usually prefer the cognate codon AAC, however, *C. albicans *had a strong preference for AAU codons (RSCU = 1.435) over AAC (RSCU = 0.565), (Additional file 2, Table S1). That is, its most frequently used Asn codon is decoded by a near-cognate tRNA (tRNA^Asn ^_GUU_). This near-cognate decoding preference was also observed for the codons corresponding to the amino acids His (**CAC**, CAU), Asp (**GAC**, GAU), Gly (**GGA**, **GGC**, **GGG, **GGU), Tyr (**UAC**, UAU), Cys (**UGC**, UGU) and Phe (**UUC**, UUU), where the preferred codons in *C. albicans *(underlined, above) do not have cognate tRNAs (codons with cognate tRNAs are indicated in bold).

**Table 4:** tRNA gene copy number *vs *total number of codons in fungi

	tRNA gene copy number	total codons
*A.fumigatus*	163	3842897
***C.albicans***	**131**	**2939109**
*C.glabrata*	207	2525088
*D.hansenii*	205	2796378
*E.gossypii*	169	2220107
*K.lactis*	162	2397264
*S.bayanus*	274	1811749
*S.cerevisiae*	273	2804657
*S.mikatae*	251	1698131
*S.paradoxus*	200	2132879
*S.pombe*	156	2062840

Complete genome sequences of the 11 fungal species studied were used to extract tRNA sequences using tRNAscan-SE (see Methods). The total number of different tRNA gene sequences retrieved was compared with the total number of codons. *C. albicans *(bold) had the smallest set of tRNA genes, but was the second largest ORFeome.

The relative low number of tRNA genes in *C. albicans *suggested that, either *C. albicans *regulates expression of certain tRNAs through yet unknown cis-acting elements and uses novel polIII transcriptional activators (i.e., tRNA gene copy numbers are not indicative of tRNA availability), or its mRNA translation machinery works under tRNA limitation. In order to clarify these important points we have scanned the 5'-upstream sequences of the *C. albicans *tRNA genes and searched for conserved elements that could explain tRNA up-regulation by the polIII transcriptional machinery. However, we were unable to identify such putative conserved polIII enhancers (data not shown). Therefore, one is left with the intriguing possibility that tRNA limitation and generalized near-cognate decoding may yet be another unique feature of the *C. albicans *translational machinery. This may explain the strong bias of *C. albicans *codon-triplet usage since, unlike in other species, *C. albicans *maximizes the utilization of a small subset of tRNAs to decode strongly biased codons present in the triplets. This puzzling result requires experimental confirmation through *in vivo *tRNAs quantification to clarify whether tRNA limitation is a feature of the *C. albicans *translational machinery, and, more importantly, whether such putative limited tRNA availability increases decoding error [17,24,25]. We have discovered recently that ambiguous decoding of the reassigned CUG codon (serine + leucine) generates phenotypic diversity in this human pathogen [26,27] and it will be most interesting to elucidate whether *C. albicans *uses generalized mistranslation as a strategy to expose hidden phenotypic diversity. Finally, we cannot exclude that biases of codon-triplets arise from protein primary structure constraints. Indeed, our study on a genetic code alteration in *C. albicans *supports this hypothesis (see below). However, tri-peptide biases would only be relevant for this study if they were significantly different in *C. albicans *and this was not observed. Rather, the main differences between *C. albicans *and the other species were related to a limited subset of contexts with repeated codons and amino acids in consecutive positions (se bellow).

### Strings of repeated codons

**Figure 4:**
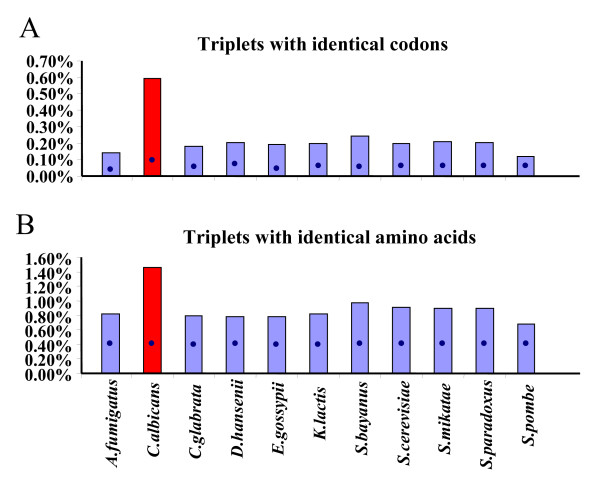
**High repetition of identical codons and amino acids in *C. albicans***. The percentage of ORFeomes composed of identical codon-triplets was determined. The percentage of these triplets (panel A) and of their respective amino acids (panel B) was much higher in *C. albicans *than in the other species, indicating a strong bias in the distribution of repeated codons in its ORFeome (red bars). Bars represent the observed percentages while blue dots indicate expected values.

**Figure 5:**
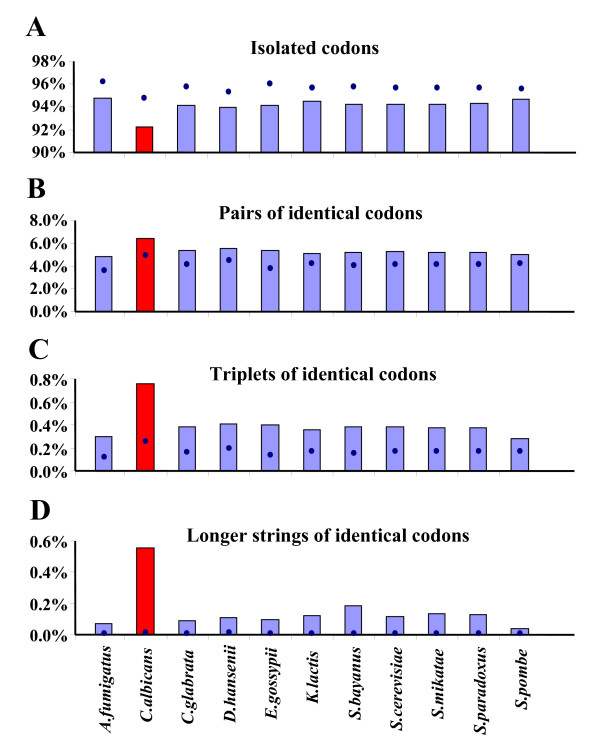
**Low frequency of isolated (non-repeated) codons in *C. albicans***. Since codon repeats were very frequent in *C. albicans *and also in the other species we have computed separated the proportion of codons that appeared isolated or in identical codon-pairs, -triplets or longer strings. *C. albicans *had lower frequency of isolated codons (non-repeated identical codons) than the other species, although there was general repression of isolated codons in all species (panel A). This bias was reversed for repetitions of 2 or more identical codons, which again was exacerbated in *C. albicans *(red bar; panels B-D).

The high frequency of repeated codons and amino acids in fungal ORFeomes and the high percentage of triplets of identical codons and amino acids in *C. albicans *(Figure 4A, B), prompted us to carry out a more detailed analysis of the codon composition of such repetitions. For this, the distribution of isolated codons, identical codon-pairs, identical codon-triplets and identical codon-strings were determined (Figure 5). Isolated codons were underrepresented in all ORFeomes, in particular in *C. albicans *(Figure 5A). However, this effect was minimized in pairs of identical codons, where observed and expected (random distribution) values were similar (Figure 5B). In *C. albicans*, the strong under-representation of isolated codons was not visible in identical codon-pairs (Figure 5B), and that bias was reversed and sharply increased for repetitions of identical codon-triplets and identical codon-strings (Figure 5C, D). Indeed, the distribution of the latter was remarkably different between *C. albicans *and the other ORFeomes (Figure 5D).

**Figure 6:**
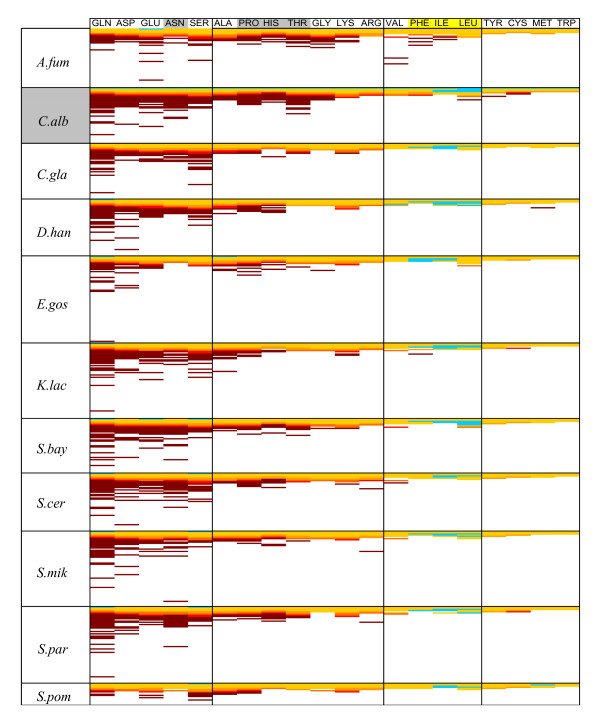
**Specificity of amino acid repeats**. The degeneracy of the genetic code prompted us to determine whether amino acid repeats would provide a better picture of the frequency of repeated features in the fungal ORFeomes. For this, the repeats were quantified and displayed as shown. In the diagram, and for each species, the first line in each column from the top corresponds to cases in which the amino acid appeared isolated in ORFs. The second line corresponds to isolated pairs of identical amino acids and so on, so that, for each column, higher number of lines correspond to longer amino acid strings. As expected, amino acid repeats were biased, as indicated by the color scale used in the map, where light blue corresponds to repressed repeats and the brown color indicates preferred repeats. Yellow represents repeats whose observed and expected frequencies were similar. Amino acid repeats were amino acid specific. For example, Gln, Asp, Glu, Asn or Ser (first group on the left) formed long strings more frequently than expected (brown), while strings of Phe, Ile, and Leu were repressed in all ORFeomes (blue bars). *C. albicans *had the longest strings of almost all amino acids, in particular of Asn, Pro, His and Thr (highlighted in grey, top of the diagram).

We then analyzed the amino acid composition of the repeated codon-triplets and again strong biases were observed (Figure 6). Most repetitions involved the amino acids Gln, Asp, Glu, Asn and Ser, confirming previous observations in *S. cerevisiae *and higher eukaryotes [28,29]. Repetitions of the amino acids Ala, Pro, His, Thr, Gly, Lys and Arg had an intermediate representation, while those of Val, Phe Ile and Leu were often underrepresented and in certain ORFeomes repetitions of Tyr, Cys, Met and Trp were absent or rarely used (Figure 6). Of all amino acids, Gln was more frequent and was also rarely present as an isolated amino acid across all ORFeomes (blue bars, first column of Figure 6). Once more, *C. albicans *showed stronger bias for amino acid repetitions since the number of Gln, Asp, Glu, Asn and Ser repetitions was higher than in the other ORFeomes, while repetitions of Pro, His and Thr, which were not frequent in the other genomes, were frequent in the *C. albicans *genome (Figure 6).

Finally, the distribution of the above repetitions was analyzed for synonymous codons of each amino acid (Additional file 1, Figures S3A,B). Of the most frequent amino acid repetitions, Gln used CAA codons mainly in all but *A. fumigatus*. Strings of Asn often used AAC codons, but some ORFeomes preferred AAT codons (Additional file 1, Figure S3A). Of the 6 serine codons, AGT, TCA and TCT were the most commonly used, while Thr repetitions used ACA or ACT but rarely ACC or ACG codons. The few Lys repetitions observed used AAG codons almost exclusively and repressed AAA codons, an effect that may be linked to strong repression of homopolymeric strings, since repetitions of CCC, GGG and TTT codons were also strongly repressed (Additional file 1, Figure S3B).

### Composition of codon-triplets that vanished from ORFeomes

**Figure 7:**
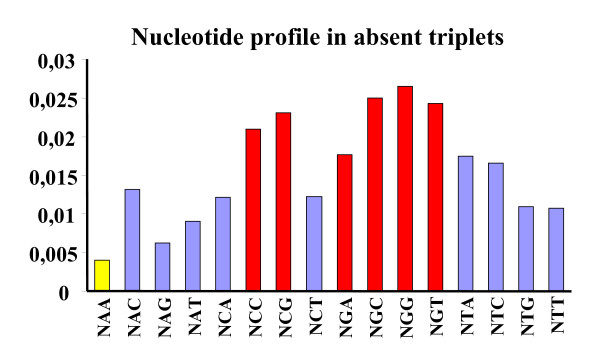
**Bias of codon-triplets that vanished from ORFeomes**. The number of possible codon-triplet combinations that were not present in fungal ORFeomes was surprisingly high. In order to elucidate why these triplets disappeared from ORFeomes, the respective codons were further studied, namely by counting the number of times each codon appeared in the first, second or third position of the triplets. No significant differences were found between species and between codon-triplet positions. Also, the first base of all codons originated redundant results. Therefore, values for all ORFeomes, triplet positions, and also for all codons starting with A, C, G or T were averaged. NCC, NCG and NGN codons were the most frequent codons in codon-triplets that were absent in fungal ORFeomes (red bars in panel B). Conversely, NAA codons were underrepresented in this group (yellow bar).

The high proportion of triplets that vanished from fungal ORFeomes (Figure 2A) prompted us to investigate whether particular codon-context trends could be identified, which would explain repression of particular codon combinations. For this, the codon composition of triplets absent in each ORFeome, at the 3 different codon positions (XXX-YYY-ZZZ), was determined. No significant differences could be detected between ORFeomes or codon positions, and the results obtained with codons starting with any base (N) were redundant (data not shown). To overcome this effect and highlight major effects only the data was averaged. A well defined pattern of preferences and rejections linked to the second and third bases of codons in absent codon-triplets (Figure 7) became apparent. This indicated that the first base of the codon, and the position of the codon in the triplet, did not contribute to triplet disappearance. Conversely, codons ending with two adenosines (NAA) showed poor association with absent triplets, while NCC, NCG or NGN were strongly associated to repressed codon triplets (Figure 7).

As before, CTG reassignment in *C. albicans *and *D. hansenii *prompted us to investigate whether the unusual decoding of CTG codons forced the disappearance of codon-triplets. For this, absent triplets that contained CTN codons, i.e. CTA, CTC, CTG or CTT were studied (Additional file 1, Figure S4). Each species had its preference pattern. The CTA codon was absent mainly in triplets of *A. fumigatus*, CTC in *C. glabrata *and *Saccharomyces *sp. and CTT in *E. gossypii*. Significantly, CTG codons were the most frequent CTN codons in codon-triplets that vanished from *C. albicans *and *D. hansenii *ORFeomes (Additional file 1, Figure S4A). Moreover, in *D. hansenii *the number of codon-triplets that vanished only from that ORFeome and lacked CTGs was two fold higher than in the other fungi, including *C. albicans *(Additional file 1, Figure S4B).

### Genetic code alteration signature

In *C. albicans *and *D. hansenii *leucine CTG codons are decoded as serine [20-22]. This genetic code alteration appeared approximately 272 ± 25 million years ago in the yeast ancestor and reprogrammed more than 30,000 CTGs present in its genes [30]. Such dramatic genetic event imposed negative pressure on CTG usage and eliminated most of these codons. Interestingly, a high number of "old" leucine-CTGs were replaced by "new" serine-CTGs that evolved from mutation of serine rather than leucine codons [30]. In other words, the CTGs existent in the ORFeomes of *C. albicans *and *D. hansenii *are new serine codons that appeared during the last 272 ± 25 million years. Since serine codons are often present in codon repetitions while leucine codons are strongly repressed (Figure 6), we have taken advantage of this genetic code alteration to shed new light on the evolutionary dynamics of codon (amino acid) repetitions in yeasts. Furthermore, since leucine is hydrophobic and serine polar, we hypothesized that constraints imposed by protein structure would be visible as alterations in the context of CTG containing triplets.

**Figure 8:**
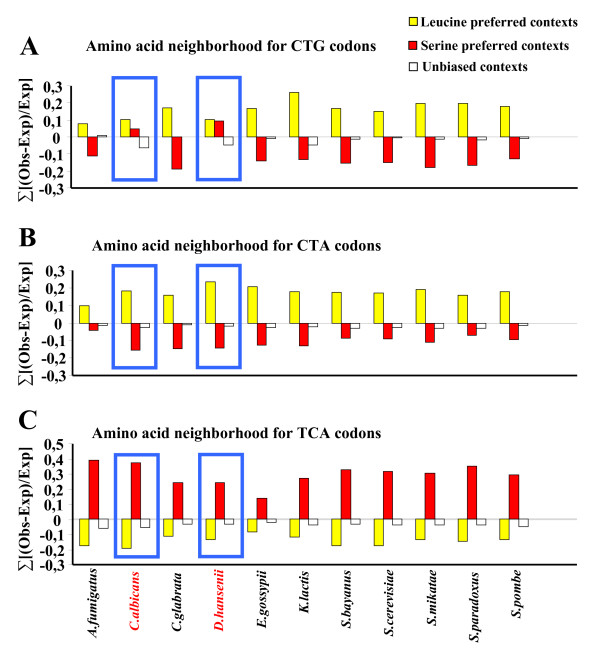
**Amino acid context signatures detect genetic code alterations**. In order to determine whether genetic code alterations could originate a specific triplet signature, the frequencies of amino acid contexts having leucine or serine in the middle position (ex. LYS-**LEU**-ASP/LYS-**SER**-ASP) were subtracted. Whenever this difference was higher than 0.0005 or lower than -0.0005 the respective context was considered biased towards leucine or serine, respectively. These biased neighborhoods were checked for Leu/Ser-CTG, Leu-CTA and Ser-TCA codons. The expected values were calculated for all the contexts and subtracted from the observed values. In order to normalize the bias with the total pool size for each codon-context each difference was divided by the expected value [(Obs-Exp)/Exp]. The sum of the quotients of all leucine-preferred (yellow bars) and serine-preferred (red bars) neighborhoods for each ORFeome showed the global effect. As expected, leucine CTG codons (panel-A) were more frequent in leucine-preferred contexts (yellow bars) than in serine-preferred ones (red bars). However, this signature was broken in *C. albicans *and *D. hansenii*, (where CTGs are decoded as serine and not leucine) since CTGs were associated with serine- rather than leucine-preferred neighbors. This trend was not detected in any other leucine or serine codon (Ex: Leu-CTA and Ser-TCA, panels-B and -C, respectively), indicating that genetic code alterations can be detected through codon-triplet context analysis.

Contexts of the NNN-Leu-NNN and NNN-Ser-NNN types were identified in the ORFeomes set and the values were displayed in such a way that upstream and downstream rejected and preferred codon neighbors could be highlighted (Figure 8). This was carried out by determining codon neighbor combinations (upstream and downstream) that were preferred in leucine- or serine-bearing triplets (leucine and serine neighbor signatures) and computing the number of times each signature appeared above the expected threshold, when the middle codon of the triplet was CTG (Figure 8A). As expected, leucine and serine had clear neighborhood preferences, but this context signature was lost for CTGs in *C. albicans *and *D. hansenii *(blue boxes, Figure 8A), which decode leucine-CTG codons as serine. In these species, CTGs had a signature that was not observed for leucine-CTA or serine-TCA codons, used as external controls (Figure 8B, 8C).

## Discussion

### Context of codon-triplets

The close phylogenetic relationship of the fungi used in this study supports the hypothesis that, like codon-pair contexts, codon-triplet contexts are species specific. If codon-triplet contexts fine tune ribosome decoding efficiency, as we have hypothesized, then it is likely that the translation machinery of these fungal species imposes different pressure on codon-triplet context. This is in line with the finding that overexpression of genes in heterologous hosts is sometimes remarkably difficult to achieve due to differences in codon usage and other yet poorly understood translational constraints [31,32]. Moreover, the fidelity of heterologous protein synthesis is often affected by codon-pair context [31], which is also species specific [33], and, since the ribosome has 3 tRNA binding sites, one would expect that codon-triplet context specificity is indeed required to fine tune mRNA decoding efficiency.

Despite the species specificity found, common trends of codon-triplet context were observed in fungal ORFeomes. For example, CC- and CG-ending codons and codons containing guanosine in the middle position, i.e. NGN (Figure 7, red bars) were repressed in codon-triplets. Conversely, codons ending with two adenosines, i.e. NAA, were rare in codon-triplets that vanished from these fungal ORFeomes (Figure 7, yellow bar). The position of each codon in the triplet and the nature of the nucleotide at the first codon position, which strongly influenced codon-pair context [4] were not relevant in vanished codon-triplet contexts. Conversely, the last and middle nucleotides of each codon influenced those codon-triplet contexts (Figure 7). Variation in the last position of codons produced most codon usage biases because nucleotide changes at this position are often silent [34], but changes in the middle position of codons frequently result in amino acid changes in protein sequences [35]. Therefore, the apparent role of the third codon position on codon-triplet context may be linked to codon usage bias, while the role of the middle nucleotide of codons may be related to protein structure constraints. When the later constraint was removed from our data set by considering synonymous codons only (Additional file 1, Figure S4), C + G pressure (*A. fumigatus *and *K. lactis*) and the *C. albicans *and *D. hansenii *genetic code alteration appeared as important modulators of codon-triplet context. Indeed, G + C pressure seems to be the reason for the enrichment of vanished codon triplets in codons ending with A or T in *A. fumigatus *and in G or C in *K. lactis *(Additional file 1, Figure S4A), a result which is inline with the global GC% of both ORFeomes (GC% = 54,01 and GC% = 40,10, respectively). In *C. albicans *and *D. hansenii *repression of triplets containing CTG codons was clearly visible, indicating that the genetic code alteration increases discrimination of CTG-associated contexts. Codon-triplets that were absent in *D. hansenii *only (Additional file 1, Figure S4B) contained twice the number of CTG-bearing codon-triplets, suggesting that non-standard decoding of CTG as serine had stronger impact in *D. hansenii *than in *C. albicans *ORFeomes.

### Strings of repeated codons

Apart from codon-triplet contexts, our software tools detected strings of repeated codons (Figures 4, 5). Tandem codon repeats are frequent in eukaryotic protein coding DNA [29] and result from slippage of the DNA polymerase δ during genome replication [36]. Such repetitions are also present in non-coding DNA in the form of trinucleotide repeats and, therefore, are unrelated to codon decoding by the ribosome [28,37]. Indeed, they are much more frequent in non-coding sequences, which contain greater variety of tandem repeats (especially from 1–6 bp) [37]. Despite this, one was prompted to ask whether tandem codon repeats could have a negative impact on decoding fidelity. For example, could ribosome frameshifting and drop off increase at codon strings due to depletion of tRNAs during decoding of repeated codons? If so, translation of the very high number of codon-strings in the *C. albicans *genome (Figure 5) would be problematic. This hypothesis was supported by the low relative abundance of tRNAs necessary to decode such repeated codon-strings (Figure 3). Indeed, of the 10 most preferred codon-triplets in *C. albicans*, 7 corresponded to contexts of repeated codons (Table 2) whose decoding involves low abundance tRNAs (either cognate or near-cognate), as, for example, the above mentioned Asn codons, AAC and AAT.

The amino acids involved in formation of codon-strings in *C. albicans *and other organisms were identical, namely Gln, Asp, Glu, Asn and Ser [28,29] (Figure 6). However, in *C. albicans *Pro, His and Thr also formed repetitions that were not observed in other organisms. Also, there was codon discrimination within amino acid repetitions (Additional file 1, Figure S3A,B). For example, in almost all ORFeomes studied Gln-CAA was more frequent than expected while its synonymous Gln-CAG was repressed (Additional file 1, Figure S3A). In *C. albicans*, Thr-ACA and Thr-ACT codons were frequently used in Thr-strings, while the Thr-ACC and Thr-ACG codons were not (Additional file 1, Figure S3A). This preference for certain codons within amino acid runs, suggests bias in DNA polymerase δ slippage or, alternatively, identical codon-repetitions produced during genome replication were later *polished *at the 3^rd ^codon position by positive pressure arising from the translation process.

Finally, in all ORFeomes studied, acidic amino acids were present more often than basic amino acids in amino acid runs, the hydrophobic amino acids Phe, Ile or Leu did not form repetitions (Figure 6) and runs of amino acids formed by homopolymeric codon strings, i.e. AAA, CCC, GGG or TTT (Additional file 1, Figure S3B) were also strongly repressed, as already observed in other eukaryotic genomes [29,37]. Since the latter corresponded to frameshifting-prone contexts [8,10,38] it is likely that their repression is related to translation fidelity. For example, the A AAA AAG motif found in *dnaX *and in many insertion sequences of the IS3 family has been considered the most efficient heptameric -1 shift motif in *E. coli *[10].

### Leucine vs serine context signatures

*C. albicans *and *D. hansenii *non-standard serine-CTG and standard leucine-CTG codons of the other fungal ORFeomes had divergent triplet-context preferences (Figure 8). The Ser-CTG codons of *C. albicans *and *D. hansenii *had codon neighbors typical of serines rather than leucines, indicating that these residues have clear neighbor preferences (upstream and downstream) and that the alteration of identity of the CTG codon from leucine to serine re-shaped the context of the codon-triplets containing CTGs. This implies that sense-to-sense genetic code alterations are accompanied by alteration in the context (upstream and downstream) of the codons that change identity to maintain amino acid triplet signatures (amino acid context). This may minimize the negative impact of genetic code alterations on protein structure and indicates that triplet amino acid context signatures are efficient tools to predict genetic code alterations.

## Conclusion

Our methodology to study codon-triplet context permitted carrying out large scale comparative analyses of triplets in 11 fungal genomes. Like codon-pair context, codon-triplet context is biased and such bias is maximal in the main human pathogen *C. albicans*. The data unveiled the nature and extent of codon repetitions in fungal ORFeomes and identified important differences in codon repetitions between fungal species. *C. albicans *showed the highest frequency and the longest codon repetitions and used codons in some repetitions that were not found in other fungi. Interestingly, codon-triplet contexts had specific signatures that were not observed for the CUG codon, which was reassigned from leucine to serine in *C. albicans *and *D. hansenii*. Such signatures highlight genetic code alterations in newly sequenced genomes.

## Methods

### Three-codon contexts

ORFeome sequences were retrieved from NCBI Genbank, the Broad Institute and from the *Candida *Genome database (Table 1). ORF sequences that did not start with the ATG codon, did not end with one of the 3 stop codons inframe (TAA, TGA, TAG) or had internal stop codons or undefined bases (N), were discarded from the dataset. For data processing, we have developed an algorithm that fixes the frame at the initiation codon (ATG) and reads the 3 first inframe codons (reading window). It then moves the reading window one codon at a time in the 3'direction and memorizes all triplets until it encounters a stop codon. The algorithm reads entire ORFeomes but discards the first and last 3 codons which have specific contexts necessary for efficient translation initiation and termination (e.g. [39]). The results obtained were stored in a tri-dimensional array of 61 × 61 × 61 dimension, which was represented by cod(i, j, k), where i, j and k were codons of the first, second and third position, respectively (Figure 1). The values stored in the array corresponded to the number of times that a particular triplet appeared in one ORFeome. Similarly, a matrix of 20 × 20 × 20 dimension was built for amino acid triplets.

**Figure 9:**
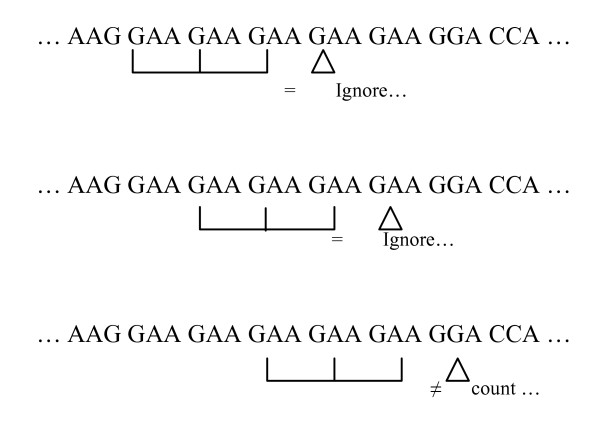
**Methodology to remove regions of tandem codon repetition**. Repetitions of more than 3 consecutive codons were excluded from the analysis by analyzing four consecutive codons at each step. At each iteration, the presence of identical codons forming 4 consecutive triplets was verified and when such triplets were found the algorithm proceeded reading without counting the triplets until a different codon appeared in the ORF sequence.

Since tandem codon repetitions were prominent in all genomes and introduced noise in the codon-triplet analysis, repetitions of more than 3 consecutive codons were excluded from the analysis during a second round of data processing. For this, the algorithm was modified as illustrated in Figure 9. The above methodology was also used for amino acid triplet counting. However, the ignored triplets present in strings were counted separately to evaluate the composition and length of each amino acid (codon) string. The results obtained for each ORFeome were stored as an array of *m *× 61, where *m *represents the maximum string length found in that ORFeome and the stored values correspond to the number of times each codon or amino acid appeared in sequences from 1 to *m*. The data arrays built by the algorithms described above were stored in a database to facilitate subsequent data analysis, which was performed using the Weka-3 package for data mining [19] and direct queries to the database (Figure 1).

Final results are shown together with expected non-biased results. To calculate the latter, we used the frequency of the respective codons or amino acids in the total ORFeome, which corresponds to the probability of their random appearance in each ORFeome. So, the expected frequency for any 3-codon context, codon1-codon2-codon3, would be the product of the frequencies of the individual codons, F(codon1)*F(codon2)*F(codon3).

### tRNA genes

tRNA genes were identified using the tRNAscan-SE software package for tRNA identification and gene copy number quantification [40]. This freeware software was used as a standalone platform, which we have modified slightly to scan several genomes automatically. The gene copy number of each tRNA isoacceptor was calculated and compared to the total number of cognate codons present in coding sequences for each species. This provided a relative measure of tRNA availability in each organism. For this, the relative synonymous codon usage (RSCU) values were calculated for all codons, according to Sharp and Li [23]. Briefly, the RSCU of a codon (X) represents the number of times it appears in a sequence (observed usage), divided by the expected usage value, assuming random usage of synonymous codons for the corresponding amino acid (C). Therefore, RSCU values for a group of synonymous codons are similar if there is no codon usage bias, but become divergent when there is codon usage bias. Since there is a strong relationship between codon usage and tRNA abundance in bacteria and in *S. cerevisiae *[2], we have used a new index to determine whether such relationship was maintained in the fungal genomes under study. For this, we have calculated the "relative isoacceptor usage" (RIU) values for all tRNAs, through the methodology below:

$${\text{RIU}}_{\text{i},\text{j}}=\frac{{\text{X}}_{\text{i},\text{j}}}{\frac{1}{{\text{n}}_{\text{i}}}\sum_{\text{j}=1}^{{\text{n}}_{\text{i}}}{\text{X}}_{\text{i},\text{j}}}$$

where *X*_*ij *_is the tRNA gene copy number for the *jth *anticodon for the *ith *amino acid, and *n*_*i *_is the number of isoacceptors for the same amino acid. We assumed that tRNA abundance is directly proportional to tRNA gene copy number, as is the case in *S. cerevisiae *and other eukaryotes [41]. As for RSCUs, RIU values for each group of isoacceptors are similar when tRNA gene copy is not biased and different when tRNA gene copy number for each tRNA isoacceptor is biased.

Finally, a decoding adaptation quotient (DAQ), which quantified the relationship (adaptation) between codon usage and tRNA abundance, was calculated by dividing RSCU by RIU values of cognate codon/tRNA pairs (DAQ = RSCU/RIU). DAQ values of 1 indicate a perfect match between tRNA copy number and codon usage, while a DAQ>1 indicates that highly used codons (high RSCU) are decoded by tRNAs whose gene copy number is low (low abundance; low RIU), and DAQ<1 indicates that codons that are used less frequently (low RSCU) are decoded by abundant tRNAs (high gene copy number; high RIU).

## Authors' contributions

GM participated in the design of the study, performed the statistical analysis and drafted the manuscript; JPL created the software for codon triplet and codon repeats quantification; MP performed the tRNAscan-SE analysis; LC and RMS participated in the discussion of the results; JLO participated in the design of the study and coordinated the work of JPL and MP; and MASS conceived the study, and participated in its design and coordination. All authors read and approved the final manuscript.

## Supplementary Material

Additional File 1 Supplementary figures. Supplementary figures representing: S1) histograms for codon-triplet distributions; S2) tRNAs and codon usage unbalance in *C. albicans*; S3A,B) codon repeat composition; and S4) the frequency of CTN codons in codon triplets that vanished from each ORFeome. Click here for file

Additional File 2 Supplementary table. The data provided represent the RSCU values as calculated for the *C. albicans*' ORFeome. Click here for file
