# Supplementary material for: Codon-triplet context unveils unique features of the Candida albicans protein coding genome
Source: BMC Genomics. 2007 Nov 29;8:444. doi: 10.1186/1471-2164-8-444 (PMC2244636; doi:10.1186/1471-2164-8-444)
Supplement: Additional File 1 — Supplementary figures. Supplementary figures representing: S1) histograms for codon-triplet distributions; S2) tRNAs and codon usage unbalance in C. albicans; S3A,B) codon repeat composition; and S4) the frequency of CTN codons in codon triplets that vanished from each ORFeome. [file 1471-2164-8-444-S1.pdf]

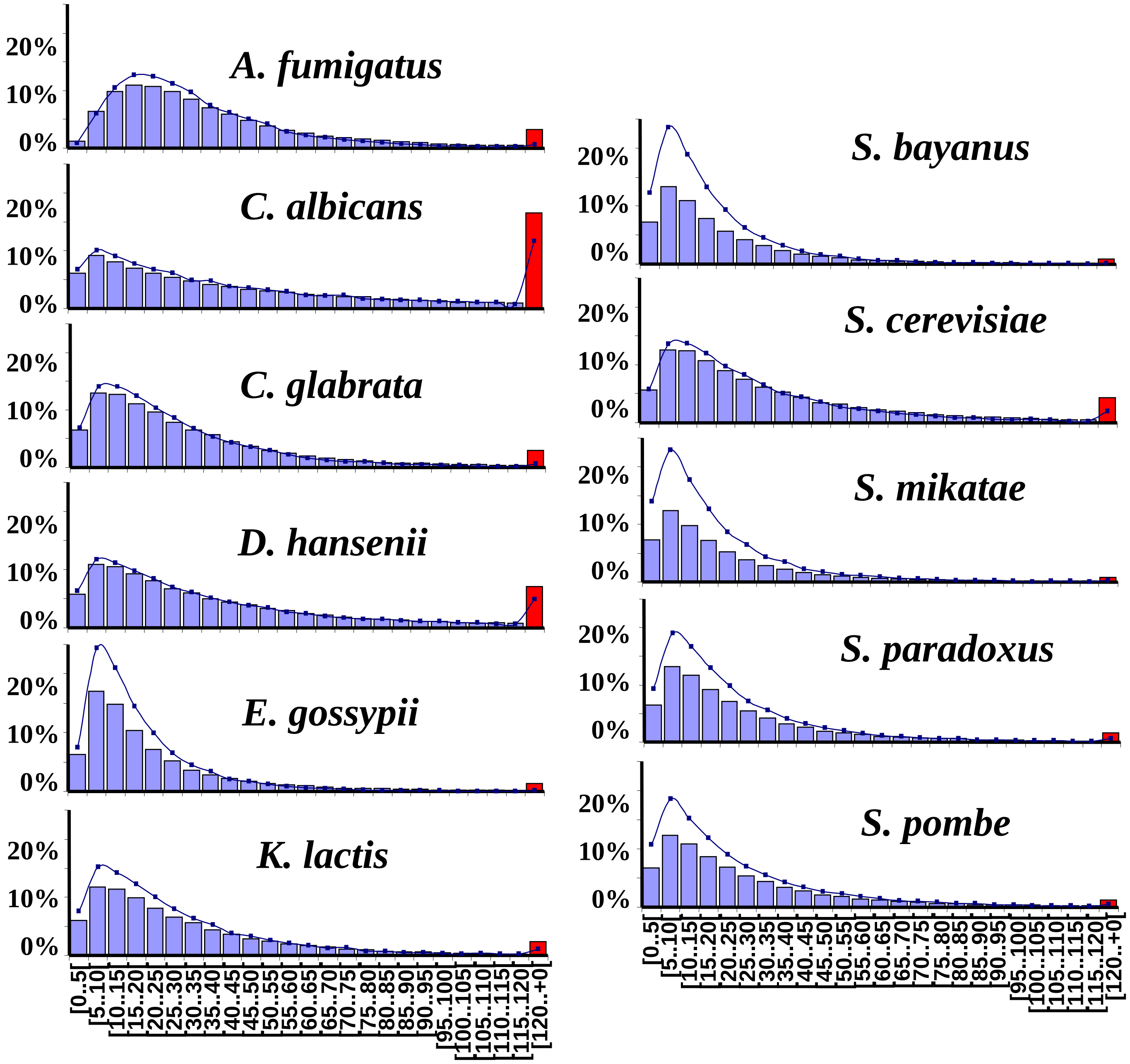

Figure S1

Three-codon contexts are species-specific. Codon-triplets were divided into 25 groups according to their usage in the ORFeomes. Observed and expected values were plotted as bars and lines, respectively. The relative size of each bar is a measure of the strength codon-triplets bias. The data shows that codon-triplets were not evenly distributed in ORFeomes since the majority was represented between 5 and 30 times (middle bars of all histograms) while few appeared more then 50 times in the same species (right half of all histograms). The *C. albicans* ORFeome originated the most divergent histogram, with very high number of frequent triplets (red bars: triplets that appeared more than 120 times in one ORFeome).

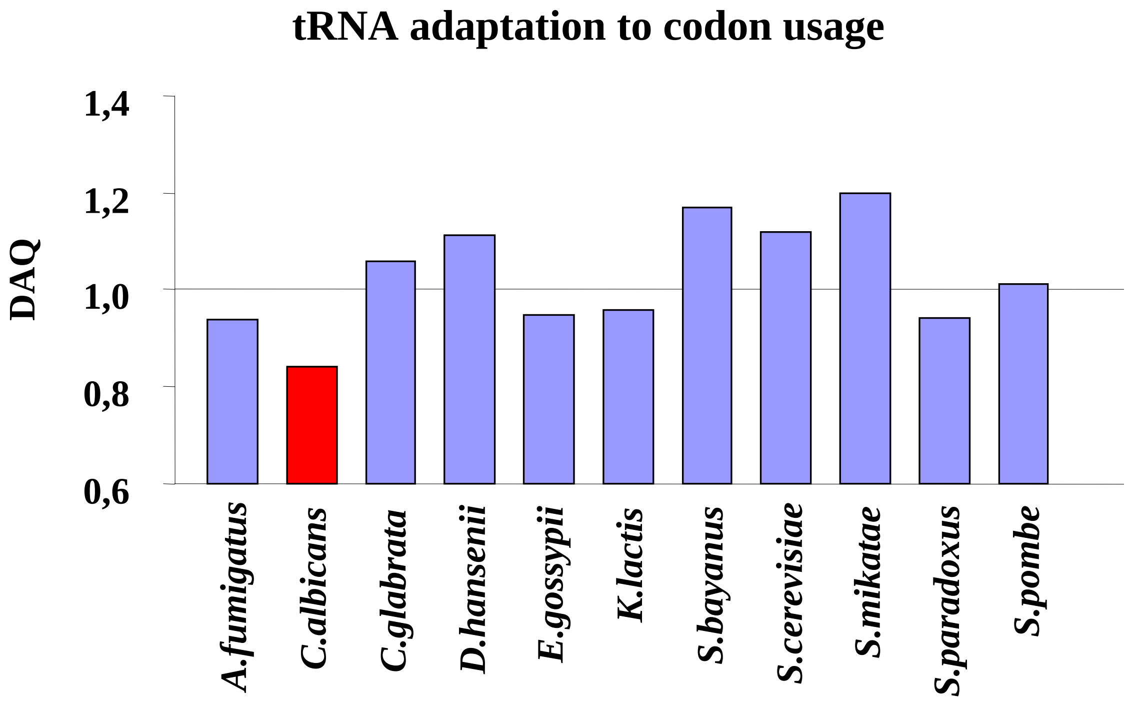

Figure S2

C. albicans **tRNAs and codon usage are unbalanced.** In order to test whether tRNAs and codon usage were unbalanced in *C. albicans*, Relative Synonymous Codon Usage (RSCU) values for all codons [24] and Relative Isoacceptor Usage (RIU) values, which measures tRNA availability, were calculated. The later was calculated as for RSCU but using gene copy number values, thus determining the fraction of isoacceptors that are utilized, i.e. the gene copy number of each tRNA, divided by the expected number assuming equilibrium between all isoacceptors for an amino acid (see Methods). The correlation between tRNA availability and codon usage is expressed in the figure, representing the Decoding Adaptation Quotient (DAQ=RSCU/RIU). DAQ values closer to 1 indicate high adaptation between tRNAs and codon usage. The *C. albicans* ORFeome was the least adapted of the 11 analyzed since it originated the lowest DAQ value. This indicates that this species *prefers* codons that are decoded by near-cognate tRNAs (see main text for a more detailed explanation).

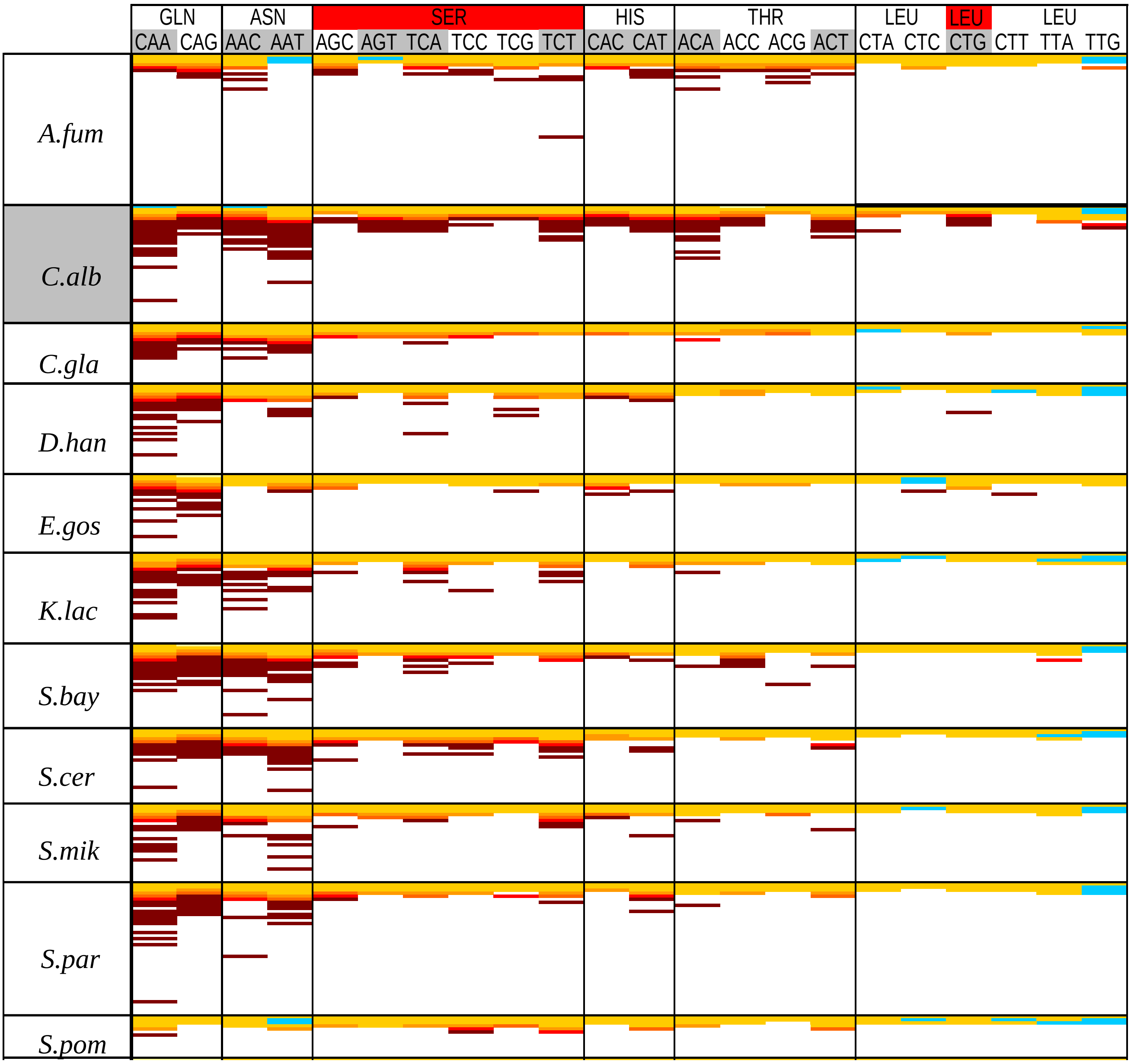

Figure S3A

Codon repeat biases are species and amino acid specific. When individual codon repeat bias was studied using the amino acid repeats methodology (Figure 6), *C. albicans* and the other species behaved differently. Both codons for one amino acid (Asn and His) or specific codons of a group of synonymous (Gln, Ser or Thr) were highly represented in long strings in the *C. albicans* ORFeome (codons highlighted in grey at the top of the diagram). Interestingly, the serine-CTG codon in *C. albicans* was also preferred in long strings, as was the case of other serine codons, but leucine codons did not form long repetitions (amino acids highlighted in red). The preference of codon repeats in *C. albicans* was mainly related with a subset of codons, namely: Gln-CAA; Asn-AAC; Asn-AAT; Ser-AGT; Ser-TCA; Ser-TCT; His-CAC; His-CAT; Thr-ACA; Thr-ACT; and Ser/Leu-CTG.

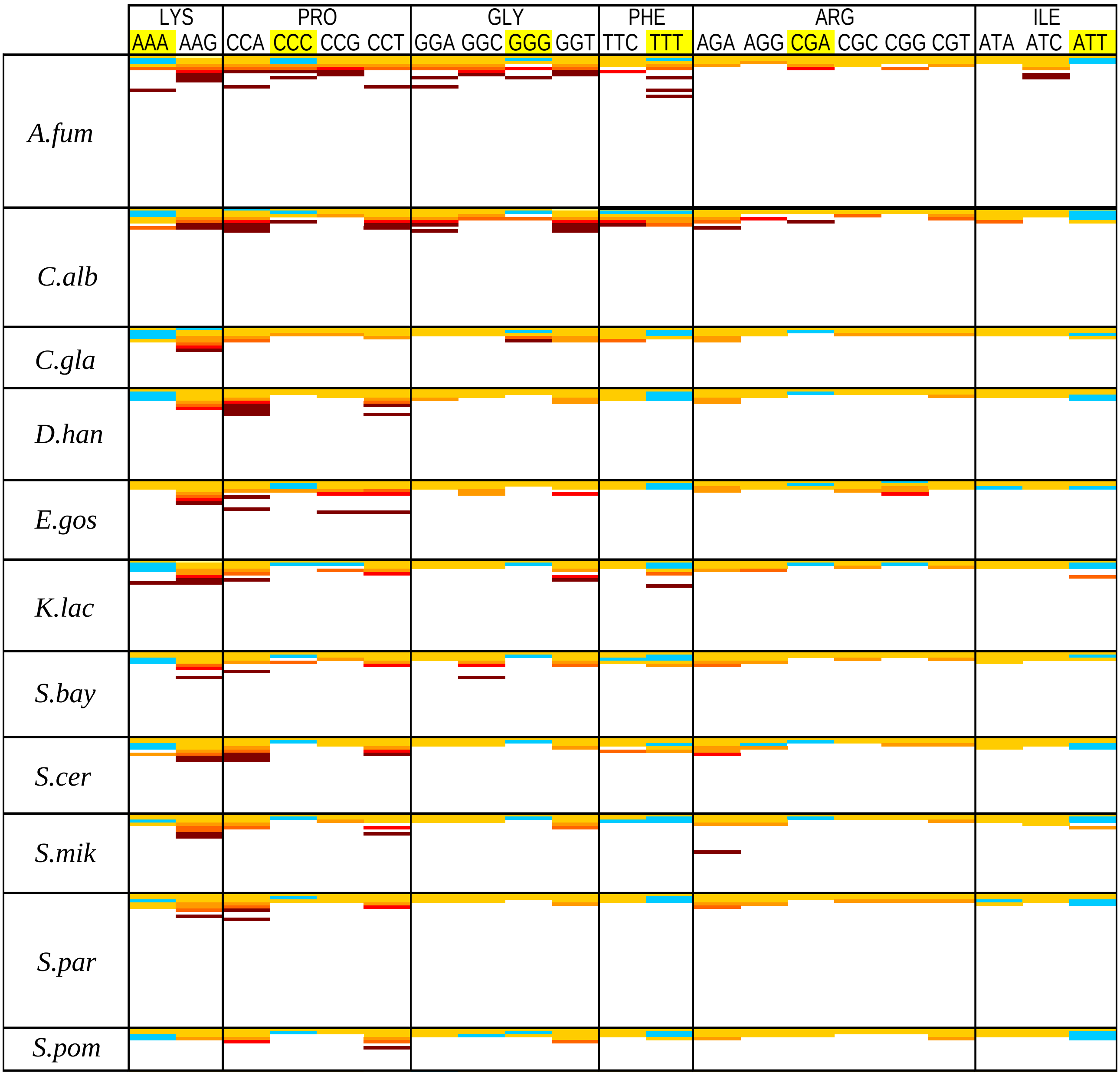

Figure S3B

Codon repeat biases are species and amino acid specific. When individual codon repeat bias was studied using the amino acid repeats methodology (Figure 6), *C. albicans* and the other species behaved differently. Amino acids whose codon repeats were repressed are shown here. These codons (highlighted in yellow at the top of the diagram) belong to families of synonymous codons that code for amino acids that are repressed also in amino acid strings (Phe, Ile, Leu – Figure 6) or are codons of homopolymeric nucleotide runs (AAA, CCC, GGG, TTT), suggesting that they are related to increased decoding error, namely frameshifting and ribosome drop-off.

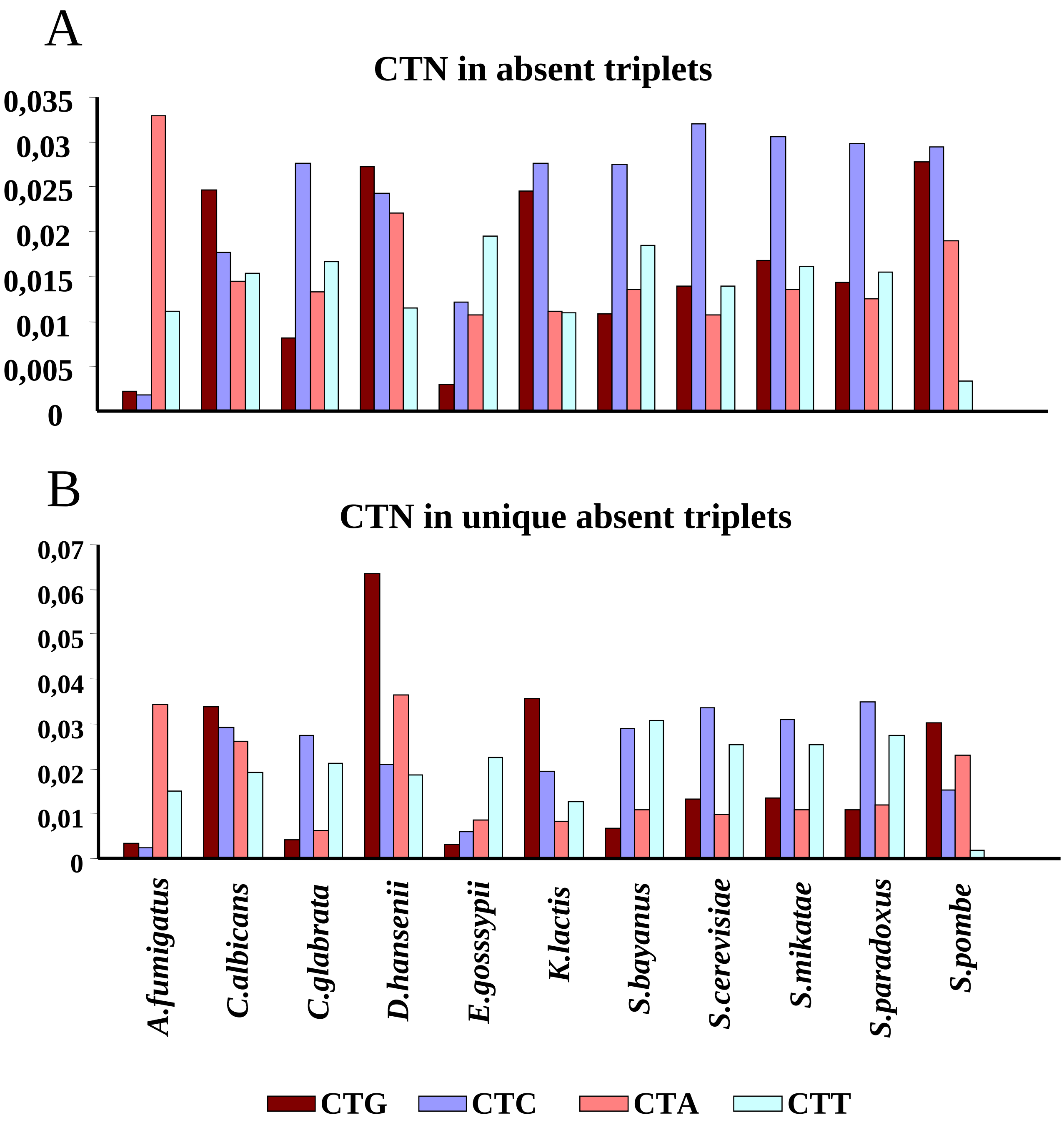

Figure S4

CTG codons in absent triplets of *C. albicans* and *D. hansenii*. Since CTG codons are decoded in two different ways among the group of 11 fungal species studied, we investigated whether its unusual decoding as serine altered its presence in triplets. For that, CTG usage in codon-triplets that vanished from ORFeomes (panel A) was calculated and compared with the result obtained for other CTN codons. Remarkably, the non-standard decoders *C. albicans* and *D. hansenii* were the only species that showed strong rejection of CTGs among the other CTN codons. The importance of CTG was maximal in triplets that vanished from *D. hansenii* ORFeome only (panel B).
