# Supplementary material for: Codon-triplet context unveils unique features of the Candida albicans protein coding genome
Source: BMC Genomics. 2007 Nov 29;8:444. doi: 10.1186/1471-2164-8-444 (PMC2244636; doi:10.1186/1471-2164-8-444)
Supplement: Additional File 2 — Supplementary table. The data provided represent the RSCU values as calculated for the C. albicans' ORFeome. [file 1471-2164-8-444-S2.pdf]

Table S1: RSCU values of *C. albicans*.

AA	codon	RSCU	AA	codon	RSCU
Lys	AAA	1,466	Trp	TGG	1,000
Lys	AAG	0,534	Leu	CTA	0,329
Asn	AAC	0,558	Leu	CTC	0,158
Asn	AAT	1,442	Leu	CTT	0,569
Thr	ACA	1,278	Leu	TTA	2,099
Thr	ACC	0,763	Leu	TTG	1,844
Thr	ACG	0,271	Pro	CCA	2,141
Thr	ACT	1,688	Pro	CCC	0,409
Arg	AGA	3,342	Pro	CCG	0,284
Arg	AGG	0,510	Pro	CCT	1,166
Arg	CGA	0,826	His	CAC	0,515
Arg	CGC	0,149	His	CAT	1,485
Arg	CGG	0,206	Gln	CAA	1,657
Arg	CGT	0,966	Gln	CAG	0,343
Ser	AGC	0,394	Val	GTA	0,721
Ser	AGT	1,389	Val	GTC	0,585
Ser	CTG	0,337	Val	GTG	0,770
Ser	TCA	2,104	Val	GTT	1,925
Ser	TCC	0,687	Ala	GCA	1,291
Ser	TCG	0,540	Ala	GCC	0,799
Ser	TCT	1,549	Ala	GCG	0,194
Ile	ATA	0,765	Ala	GCT	1,717
Ile	ATC	0,551	Gly	GGA	1,148
Ile	ATT	1,684	Gly	GGC	0,352
Met	ATG	1,000	Gly	GGG	0,598
Phe	TTC	0,638	Gly	GGT	1,902
Phe	TTT	1,362	Asp	GAC	0,449
Tyr	TAC	0,549	Asp	GAT	1,551
Tyr	TAT	1,451	Glu	GAA	1,587
Cys	TGC	0,359	Glu	GAG	0,413
Cys	TGT	1,641			

RSCU values were calculated as explained by Sharp and Li [24], using all genes of *C. albicans*. For the calculations, CTG was considered a serine codon.
